# Pembrolizumab as first-line treatment for recurrent or metastatic head and neck squamous cell carcinoma: a multi-institutional DAHANCA cohort study

**DOI:** 10.2340/1651-226X.2025.44327

**Published:** 2025-09-21

**Authors:** Sebastian Søby, Robin Prestwich, Niels Gyldenkerne, Christian Maare, Camilla K. Lonkvist, Maria Andersen, Kinga Nowicka-Matus, Anita Gothelf, Trine Tramm, Kasper Toustrup, Simion Mihaela, Danny Ulahannan, Zsuzsanna Iyizoba-Ebozue, Jesper G. Eriksen

**Affiliations:** a Department of Experimental Clinical Oncology, Aarhus University Hospital, Aarhus, Denmark; b Department of Clinical Medicine, Aarhus University, Aarhus, Denmark; c Leeds Cancer Center, St. James University Hospital, Leeds, England; d Department of Oncology, Odense University Hospital, Odense, Denmark; e Department of Oncology, Herlev Hospital, Copenhagen, Denmark; f Department of Oncology, Aalborg University Hospital, Aalborg, Denmark; g Department of Oncology, Copenhagen University Hospital, Copenhagen, Denmark; h Department of Pathology, Aarhus University Hospital, Aarhus, Denmark; i Department of Oncology, Aarhus University Hospital, Aarhus, Denmark; j Leeds Institute of Medical Research, University of Leeds, Leeds, England

**Keywords:** Pembrolizumab (C582435), clinical trials, Phase IV as topic (D017327), B7-H1 antigen (D060890), head and neck neoplasms (D006258), palliative medicine (D065126)

## Abstract

**Background and purpose:**

Pembrolizumab is frequently used for recurrent or metastatic head and neck squamous cell carcinoma (rmHNSCC) as first-line treatment. This study evaluates real-world effectiveness in a Danish and United Kingdom (UK) cohort.

**Patient/material and methods:**

Patients with confirmed rmHNSCC treated with pembrolizumab (2020–2024) as first-line palliative treatment were included consecutively. Data were collected from patient files at four Danish head and neck cancer centres (discovery cohort) and the Leeds Teaching Hospitals NHS Trust (comparison cohort). Primary endpoints were overall survival (OS) and progression-free survival (PFS).

**Results:**

In the discovery cohort (*n* = 228), a median OS of 10 months (95% [confidence interval [CI]: 10–12) and median PFS of 4 months (95% CI: 4–6) were found. Primary endpoints did not differ significantly between the discovery and comparison cohort. Baseline World Health Organization performance status appeared to negatively impact endpoints (hazard ratio: 1.4 [95% CI: 1.0–2.0]).

**Interpretation:**

In this real-world multi-centre study, pembrolizumab demonstrated efficacy equivalent to the registration studies in two independent cohorts.

## Introduction

Immune checkpoint inhibitors such as the Programmed cell Death protein 1 (PD-1) inhibitors, nivolumab, and pembrolizumab, have emerged as a promising approach in the treatment of various malignancies, including head and neck cancer [1, 2]. Despite the optimism around PD-1 inhibition in recurrent/metastatic head and neck squamous cell carcinoma (rmHNSCC), the translation of its clinical success from controlled trial settings to real-world clinical practice remains an essential question. Patients selected for clinical trials are often younger and healthier with less comorbidity and better performance than the typical patient treated for rmHNSCC in the clinic [3, 4]. To address this disparity and evaluate the efficacy in a more representative clinical setting, a national phase IV study was conducted investigating the real-world efficacy of nivolumab. The study concluded that the efficacy of nivolumab is on par with that found in clinical trials, and that nivolumab should be considered a relevant alternative to chemotherapy [4]. However, in Denmark, nivolumab has largely been replaced by pembrolizumab, with PD-1 inhibition now being offered as first-line therapy to histologically eligible patients. In this new context, the present national multi-centre study aims prospectively to assess the efficacy as well as the toxicity of pembrolizumab outside the tightly regulated confines of a clinical trial, while including an external comparison cohort to assess the generalisability of findings across diverse clinical contexts.

## Patients/material and methods

### Study design and participants

This was a retrospective multi-centre cohort study, consecutively including eligible patients prospectively from four Danish treatment centres (Aalborg University Hospital, Aarhus University Hospital, Odense University Hospital, and Herlev Hospital) between June 2020 and December 2023. As well as an external retrospective comparison cohort with patients treated at Leeds Teaching Hospitals NHS Trust (United Kingdom) between July 2020 and June 2024. Inclusion criteria were histologically and radiologically confirmed rmHNSCC and pembrolizumab administered as first-line monotherapy, to which a prerequisite was a combined positive score (CPS) PD-L1 expression of at least 1. In England, pembrolizumab is approved by the National Institute of Clinical Excellence (NICE) for first-line treatment of rmHNSCC for patients with CPS ≥1 and a World Health Organization (WHO) performance status of 0 or 1. Pembrolizumab is not approved/available to be used in combination with chemotherapy.

Initial patient identification was done using appropriate institutional databases (the DAHANCA database for the discovery cohort). This was validated with patients identified by each centre to ensure the most complete inclusion across all centres. Patient identification on site was done through cross-referencing of ICD-10 codes with the national SKS-code system describing diagnosis and treatment respectively.

Patient demographics (date of birth, WHO performance status [WHO PS], baseline neutrophil granulocyte count, and concomitant glucocorticoid treatment) and treatment details (dates for start/end of treatment, cycles administered, reason for discontinuation, and prior cancer treatment) were retrieved from medical records, through the DAHANCA database for the discovery cohort, and from an institutional database for the comparison cohort.

The effects of patient age on treatment outcomes were analysed both as a continuous and binary variable (≥ 70 vs. < 70 yrs.). Baseline WHO PS was analysed by comparing patients with WHO PS 0–1 to those with WHO PS 2–3.

The potential impact of concomitant glucocorticoid treatment was investigated (regardless of dose and indication) using the relative exposure (number of days with systemic glucocorticoids prescribed out of the total duration of pembrolizumab treatment).

Patients with baseline neutrophilia were compared with those without, as neutrophilia has previously been associated with an increased risk of progression [4]. Neutrophilia was defined as a blood neutrophil count > 7.00*10^9^/l.

### Treatment

Pembrolizumab was administered as single-agent therapy intravenously, predominantly as 2 mg/kg body weight every 21 days. Danish patients who exhibited a lasting good response to the treatment were transitioned to a double dosage regimen, with treatment administered every 6 weeks after a few months (mo) according to U.S. Food and Drug Administration (FDA) recommendations [5].

### Radiological assessment

In Denmark, routine evaluation computed tomography (CT) scans were utilised at each centre to assess treatment response (defined by iRECIST criteria [6]). These scans were radiologist interpreted and conducted at baseline and approximately every 2–3 months afterwards. In Leeds, patients were routinely scanned with CT at baseline and approximately 3 monthly intervals. Radiologists reported responses based upon objective measurements but not formally using iRECIST.

Furthermore, patients’ electronic medical records were searched to identify cases of supposed pseudoprogression by searching for keywords related to the phrase ‘ pseudoprogression’. For these identified cases, it was registered whether early radiological progression had occurred as well as whether treatment was continued based on the clinical assessment that the progression was deemed pseudoprogression.

### PD-L1

In most cases, PD-L1 expression was described using a combined positive score evaluated according to manufacturer instructions (PD-L1 22C3 pharmDx kit from Agilent Technologies, Inc.) defined as the total number of PD-L1 positive cells (tumour cells, lymphocytes, and macrophages) divided by the total number of viable tumour cells times 100. Re-evaluations were completed by an experienced pathologist (TT) if tumour PD-L1 expression was missing or described using tumour proportion score (TPS). However, re-evaluations were not possible for the comparison cohort.

The impact of pre-treatment PD-L1 scores was assessed using CPS as both a continuous variable as well as a binary with cut-off 20, as has been done previously by Burtness et al. [7].

### Toxicity

Adverse events of any grade documented in medical records as leading to discontinuation of treatment were captured. Less severe adverse events without direct treatment influence were recorded using the patient-reported outcome measure (PROM) implemented as part of patient follow-up (Ambuflex [8]) at one of the centres (Aarhus, Denmark).

### Data analysis

Descriptive statistics were employed to describe patient population, tumour, and treatment characteristics. The primary endpoint, overall survival (OS), was calculated from the first day of immunotherapeutic treatment to the date of death or study conclusion (January 2024 for the discovery cohort; July 2024 for the comparison cohort). The secondary endpoint, progression-free survival (PFS), was assessed using the same criteria, as well as time of disease progression, whichever transpired first. The overall response rate (ORR) was calculated as the fraction of patients obtaining either partial response or complete response as defined by the iRECIST criteria.

Disease control rate (DCR) was calculated in the same manner as ORR but also included patients with stable disease. Survival curves were derived through the Kaplan-Meier method. The potential impact of prognostic factors on study endpoints was evaluated using the Cox regression model. Hazard ratios (HRs) with 95% confidence intervals (CIs) were calculated to estimate the strength and direction of associations. Throughout all analyses, two-sided tests were conducted. All statistical analyses were conducted using Stata.

## Results

### Patient and treatment characteristics

The study included 228 patients in the discovery cohort and 101 in the comparison cohort. The cohorts appeared to differ only concerning baseline WHO PS (Table 1).

**Table 1:** Patient and treatment characteristics by patient cohort.

Patient and Treatment Characteristics	Denmark	Leeds	*P*
	*N* = 228	*N* = 101	
**Sex** (male)	76% (174)	81% (82)	0.3
**Primary site**			0.4
Larynx	15% (35)	10% (10)	
Hypopharynx	12% (27)	13% (13)	
Oropharynx	39% (89)	44% (44)	
Nasopharynx	3% (6)	1% (1)	
Oral cavity	23% (52)	23% (23)	
Sino-nasal	5% (12)	8% (8)	
Salivary gland	0% (0)	1% (1)	
Unknown primary	3% (7)	1% (1)	
**Age** (median + range)	67 (40–88)	67 (34–89)	0.8
**WHO PS**			< 0.001
0–1	79% (179)	100% (101)	
≥ 2	21% (49)	0% (0)	
**Neutrophilia**	34% (77)	28% (27)	0.3
**PD-L1** (≥ 20)	60% (136)	41% (37)	< 0.001
**Cycles** (median + range)	4 (1–33)	5 (1–35)	0.07
**Best response** (iRECIST*)			< 0.001
PD	25% (56)	53% (54)	
SD	37% (84)	11% (11)	
PR	15% (34)	11% (11)	
CR	4% (9)	1% (1)	
N/A	20% (45)	24% (24)	
**Cause of discontinuation**			0.3
Progressive disease	53% (121)	52% (53)	
General condition	23% (53)	28% (28)	
Adverse events	16% (37)	12% (12)	
Response	3% (6)	0% (0)	
Still in treatment	5% (11)	8% (8)	
**Pseudoprogression**			
Not considered	88% (200)	N/A	
PD → PD	9% (21)	N/A	
PD → SD/PR/CR	3% (7)	N/A	

* iRECIST stages: PD: progressive disease; SD: stable disease; PR: partial response; CR: complete response.

Treatment characteristics only differed significantly in terms of best treatment response, where more patients in the Leeds cohort had progressive disease. Data on pseudoprogression were only available for the Danish cohort.

### Overall efficacy

**Figure 1:**
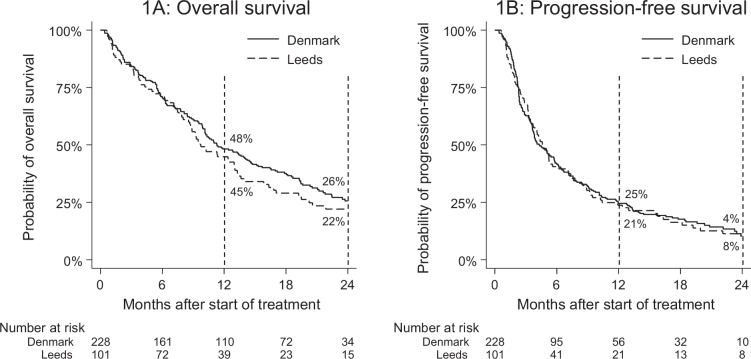
Overall survival (1A) and progression-free survival (1B) by patient cohort.

In the Danish discovery cohort, the median OS for pembrolizumab was 10 mo (95% CI: 10–12) (Figure 1A) with a 1 and 2 year survival of 48 and 26%, respectively. The median PFS was 4 mo (95% CI: 4–6) (Figure 1B) with a 1 and 2 year PFS of 25 and 4%, respectively. In this cohort, we observed an ORR of 19% (95% CI: 14–24) and a DCR of 56% (95% CI: 49–62).

In the Leeds comparison cohort, a median OS of 10 mo (95% CI: 9–13) was found with a 1 and 2 year survival of 45 and 22%, respectively. In this cohort, the median PFS was 5 mo (95% CI: 4–6) with a 1 and 2 year PFS of 21 and 8%, respectively. Furthermore, we observed an ORR of 16% (95% CI: 9–26) and a DCR of 30% (95% CI: 21–41).

Comparing patients based on whether they were treated within the approved head and neck pembrolizumab regimen (larynx, pharynx and oral cavity vs. sino-nasal, salivary gland and unknown primary) showed no significant difference in terms of OS or PFS (HR_OS_: 0.7 [95% CI: 0.5–1.2], HR_PFS_: 0.9 [95% CI: 0.6–1.4]).

### Toxicity

Adverse events that led to discontinuation of immunotherapy occurred in 16% of patients in the discovery cohort and 12% in the comparison cohort. Table 2 presents the toxicities leading to treatment discontinuation, and no significant difference was found between the two patient cohorts. The most commonly occurring adverse event overall was hepatitis, occurring in 4% of patients in the discovery cohort and 2% in the comparison cohort. Only 45 (20%) Danish patients had at least one PROM entry with a median entry count of 5 (range: 1–36) (Supplementary material #1).

**Table 2:** Adverse events, which led to discontinuation of immunotherapeutic by patient cohort.

Grade III-IV Events	Total	Denmark	Leeds	*P*
	*N* = 329	*N* = 228	*N* = 101	
**Adverse event**				0.3
Hepatitis	4% (12)	4% (10)	2% (2)	
Colitis	3% (10)	2% (5)	5% (5)	
Pneumonitis	2% (7)	3% (7)	0% (0)	
Skin reaction	2% (6)	2% (4)	2% (2)	
Arthralgia/myalgia	1% (5)	2% (4)	1% (1)	
Diarrhoea	1% (3)	1% (3)	0% (0)	
Pancreatitis	1% (2)	1% (2)	0% (0)	
Hypofysistis	1% (2)	1% (2)	0% (0)	
Fatigue	0% (1)	0% (1)	0% (0)	
Headache	0% (1)	0% (1)	0% (0)	
Nefritis	0% (1)	0% (0)	1% (1)	
Hypothyroidism	0% (1)	0% (0)	1% (1)	
Other	1% (4)	1% (3)	1% (1)	

### Patient age and WHO performance status

Patient age at baseline (≥ 70 vs. < 70 yrs.) appeared not to influence patient survival in terms of both OS (HR_OS_: 1.1 [95% CI: 0.8–1.5]) or PFS (HR_PFS_: 0.8 [95% CI: 0.6–1.1]). Results were similar when analysing patient age as a continuous variable. In the multivariate Cox-regression analysis (Table 3), patient age remained a non-significant co-factor, whether modelled as a binary or continuous variable.

**Table 3:** Multivariate Cox-regression analysis with endpoints death and progression and the variables baseline age (70 years cut-off), WHO performance status (0–1 or 2–3), neutrophilia (> 7.00*109/l) and PD-L1 CPS (≥ 20).

Covariates	Risk of death		Risk of progression	
	HR	95% CI	HR	95% CI
**Age ≥ 70 years**	1.0	0.7–1.4	0.8	0.6–1.1
**WHO PS > 1**	2.6	1.8–3.8	1.4	1.0–2.0
**Neutrophilia**	1.7	1.2–2.4	1.7	1.2–2.3
**PD-L1 ≥ 20**	0.9	0.6–1.2	1.0	0.7–1.3

**Figure 2:**
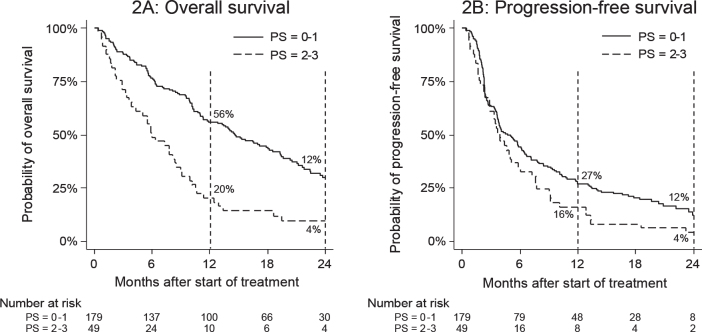
Overall survival (2A) and progression-free survival (2B) by WHO PS at baseline. WHO PS: World Health Organization performance status.

Poor performance status (WHO PS: 2-3) was strongly associated with a reduced OS (HR: 2.4 [95% CI: 1.7–3.4]) (Figure 2A) and less so with PFS (HR: 1.4 [95% CI: 1.0–2.0]) (Figure 2B). In the multivariate analysis (Table 3), WHO PS was significantly associated with an increased risk of death (HR: 2.6 [95% CI: 1.8–3.8]), but barely with an increased risk of progression (HR: 1.4 [95% CI: 1.0–2.0]). (Graphs comparing WHO PS 0 with 1 and 2–3 as well as HR for WHO PS = 0 vs. WHO PS = 1–3 can be found in Supplementary material #2.).

### PD-L1 as biomarker

**Figure 3:**
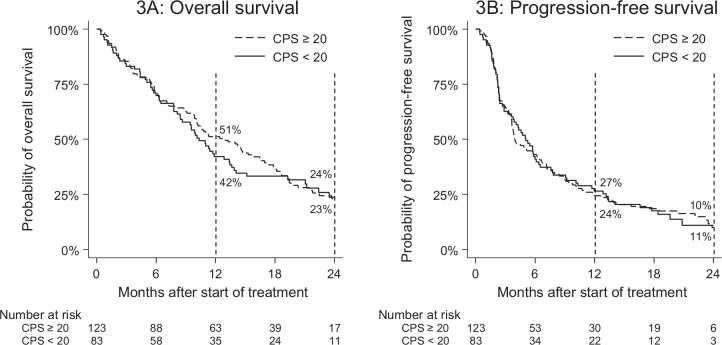
Overall survival (3A) and progression-free survival (3B) by pre-treatment CPS PD-L1 expression. CPS: combined positive score.

Pre-treatment CPS explored in the Danish cohort was not associated with OS (Figure 3A) or PFS (Figure 3B) (HR_OS_: 1.0 [95% CI: 0.7–1.4], HR_PFS_: 1.0 [95% CI: 0.7–1.3]) when using cut-off CPS 20 or when exploring CPS as a continuous variable (HR: 1.0 [95% CI: 0.99–1.0]). Furthermore, PD-L1 expression was not associated with a reduced risk of death or progression during the multivariate analysis (Table 3), regardless of whether it was modelled as continuous or binary.

### Concomitant steroid treatment & blood neutrophils

Concomitant treatment with systemic glucocorticoids was administered to 40 patients (18%) in the Danish cohort and appeared not to impact treatment outcomes (HR_OS_: 1.0 [95% CI: 1.0–1.0], HR_PFS_: 1.0 [95% CI: 1.0–1.0]).

**Figure 4:**
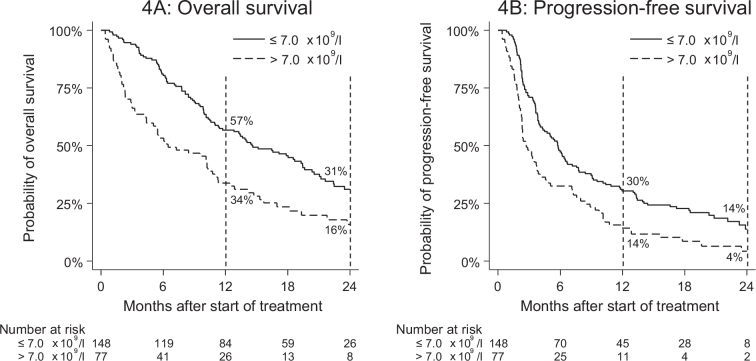
Overall survival (4A) and progression-free survival (4B) by neutrophilia at baseline.

Neutrophilia at baseline was significantly associated with both increased risk of death (Figure 4A) and risk of progression (Figure 4B) (HR_OS_: 1.8 [95% CI: 1.3–2.5], HR_PFS_: 1.8 [95% CI: 1.2–2.5]). This remained the case during the multivariate analysis (Table 3) in terms of both risk of death (HR: 1.7 [95% CI: 1.2–2.4]) and risk of progression (HR: 1.7 [95% CI: 1.2–2.3]).

### Pseudoprogression

In 28 out of 228 Danish patients (12%), early radiological progression was observed, but pembrolizumab treatment was not discontinued due to the clinically assessed possibility of pseudoprogression. Out of these 28 patients, 21 (75%) experienced progression, whereas 7 (25% [95% CI: 12–45]) went on to obtain either stable disease or partial response.

## Discussion and conclusion

In this large multi-centre Danish study, a median OS of 10 mo (95% CI: 10–12), median PFS of 4 mo (95% CI: 4–6), and ORR of 16% (95% CI: 9–26) was observed for pembrolizumab. These results are very similar to what was observed in the phase III studies, and are not significantly different from the estimates obtained by Burtness et al. [7] (mOS: 12 [95% CI: 11–14], mPFS: 3 [95% CI: 2–3], ORR: 23%). This suggests that the findings of prior phase III results apply to the average rmHNSCC clinical patient, who is often older and with worse general condition than those offered participation in the clinical trials.

No significant differences were found between the discovery and comparison cohort in terms of OS, PFS, ORR, and the frequency of each grade III–IV adverse events. This supports the hypothesis that the Danish patient cohort is comparable to other, especially Western, cohorts of similar ethnographic composition. Our findings in both the discovery and comparison cohort also align with the findings of Kong et al. [3] in a similar UK multi-centre pembrolizumab cohort (mOS: 11 [95% CI: 9–13], mPFS: 5 [95% CI: 4–6], ORR: 25%).

Pseudoprogression was observed in 25% (95% CI: 12–45%) of patients with early progression and good general condition, similar to rates observed in melanoma patients undergoing PD-1 inhibition therapy (28%) [9]. This highlights the need for careful evaluation to distinguish true disease progression from treatment-related changes, which may significantly impact therapeutic decision-making and patient management strategies.

As previously shown for nivolumab, WHO PS is hypothesised to play a major role in determining both survival and progression outcomes [4]. For pembrolizumab, in the Danish cohort, this effect appeared primarily with the endpoint OS. However, if comparing patients with WHO = 0 to those with WHO PS 1–3, a considerable impact on PFS by WHO PS becomes evident (Supplementary material #2C). Thus, WHO PS remains a key prognostic factor during PD-1 inhibition, and pembrolizumab should be prescribed with caution in patients with WHO PS > 1.

Due to ineligibility of patients with WHO PS > 1 for pembrolizumab in England, this group was not present in the comparison cohort. However, differences in treatment efficacy between the two cohorts remain non-significant while excluding the WHO PS > 1 subgroup from the discovery cohort.

Neutrophilia has been associated with an increased rate of disease progression in HNC patients undergoing treatment with nivolumab [4]. Neutrophilia is often linked to a high neutrophil-lymphocyte ratio, which is in turn associated with poor prognosis in many cancers [10]. It may reflect an imbalance in the immune response, with an excess of pro-tumour inflammatory cells and a relative depletion of anti-tumour lymphocytes [11]. In this study, neutrophilia appeared strongly associated with risk of death and progression. However, in Denmark, lymphocyte count is not routinely recorded during follow-up, which is why these results are limited to the effects of blood neutrophil level. Since both neutrophilia and WHO PS are evaluated at baseline, and these co-factors are likely to be associated, WHO PS is a possible confounder for the impact of neutrophilia on the efficacy of PD-1 inhibition.

As other researchers have also demonstrated [12], the degree of PD-L1 expression is an unreliable indicator of treatment efficacy. Our results are no different. Multiple analyses for PD-L1 expression were conducted, and no significant associations were found even without correcting for multiple testing. This highlights an urgent need for reliable prognostication and supporting predictive factors within the area of PD-1 therapy, which is accentuated by the major variance observed in treatment outcomes, where the majority of patients suffer progression before 6 months and only around 20% are progression-free after 1 year (Figure 1B). The disparity between these patients undergoing the same treatment suggests the presence of unidentified factors influencing the treatment outcome. Further research is required in this area, to enable early identification of long-term responders.

In this national cohort of rmHNSCC patients treated with pembrolizumab as first-line therapy, an efficacy and treatment-limiting toxicity similar to the registration studies were found and further compared to a separate UK cohort. Pembrolizumab monotherapy remains a relevant treatment option for patients with rmHNSCC in good performance, including those in the elderly subpopulation.

## Supplementary Material



## Data Availability

The permission from local authorities to conduct the present study does not include the option to share data – not even in a de-identified form. However, the corresponding author will be available for any other requests that may arise.
