# Supplementary material for: Pembrolizumab as first-line treatment for recurrent or metastatic head and neck squamous cell carcinoma: a multi-institutional DAHANCA cohort study
Source: Acta Oncol. 2025 Sep 21;64:44327. doi: 10.2340/1651-226X.2025.44327 (PMC12476047; doi:10.2340/1651-226X.2025.44327)
Supplement: Supplementary file 1 [file AO-64-44327-s1.pdf]

Supplementary material has been published as submitted. It has not been copyedited, or typeset by Acta Oncologica

Supplementary #1

**Adverse event**	N=45
Fatigue	71% (32)
Fever	18% (8)
Nausea	53% (24)
Diarrhea	82% (37)
Constipation	51% (23)
Abdominal pain	47% (21)
Paresthesia	58% (26)
Loss of muscle function	76% (34)
Muscle weakness	62% (28)
Skin symptoms	58% (26)
Dyspnea	56% (25)
Eye symptoms	76% (34)
Chest pain	62% (28)
Arthralgia/myalgia	49% (22)
Headache	53% (24)

Supplementary #1. Patient reported grade I-II adverse events. Displayed at as number of patients with given event of clinical severity at any time during treatment.

Supplementary #2

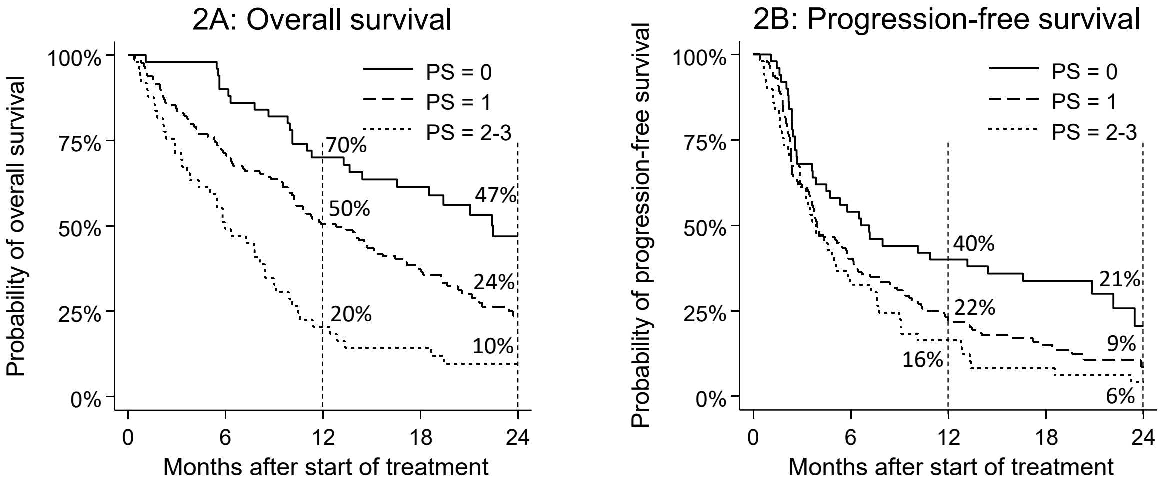

Number at risk (2A)

	0	6	12	18	24
PS = 0	50	45	35	27	13
PS = 1	129	92	65	39	17
PS = 2-3	49	24	10	6	4

Number at risk (2B)

	0	6	12	18	24
PS = 0	50	27	20	15	4
PS = 1	129	52	28	13	4
PS = 2-3	49	16	8	4	2

Supplementary #2. Overall survival [2A] and progression-free survival [2B] by baseline WHO PS 0, 1 and 2-3.

Supplementary #2C. WHO PS = 0 compared to WHO PS = 1-3:
HR_{OS}: 2.0 [95% CI: 1.4-3.0]; HR_{PFS}: 1.7 [95% CI: 1.2-2.4]
